# Contribution of GnIH Research to the Progress of Reproductive Neuroendocrinology

**DOI:** 10.3389/fendo.2015.00179

**Published:** 2015-11-23

**Authors:** Kazuyoshi Tsutsui, Takayoshi Ubuka, You Lee Son, George E. Bentley, Lance J. Kriegsfeld

**Affiliations:** ^1^Laboratory of Integrative Brain Sciences, Department of Biology and Center for Medical Life Science, Waseda University, Tokyo, Japan; ^2^Brain Research Institute Monash Sunway of the Jeffrey Cheah School of Medicine and Health Sciences, Monash University Malaysia, Petaling Jaya, Malaysia; ^3^Department of Integrative Biology, Helen Wills Neuroscience Institute, University of California at Berkeley, Berkeley, CA, USA; ^4^Department of Psychology, Helen Wills Neuroscience Institute, University of California at Berkeley, Berkeley, CA, USA

**Keywords:** gonadotropin-inhibitory hormone, gonadotropin-releasing hormone, gonadotropins, reproduction, reproductive behavior, melatonin, stress, social environment

## Abstract

Since the discovery of gonadotropin-releasing hormone (GnRH) in mammals at the beginning of the 1970s, it was generally accepted that GnRH is the only hypothalamic neuropeptide regulating gonadotropin release in mammals and other vertebrates. In 2000, however, gonadotropin-inhibitory hormone (GnIH), a novel hypothalamic neuropeptide that actively inhibits gonadotropin release, was discovered in quail. Numerous studies over the past decade and a half have demonstrated that GnIH serves as a key player regulating reproduction across vertebrates, acting on the brain and pituitary to modulate reproductive physiology and behavior. In the latter case, recent evidence indicates that GnIH can regulate reproductive behavior through changes in neurosteroid, such as neuroestrogen, biosynthesis in the brain. This review summarizes the discovery of GnIH, and the contributions to GnIH research focused on its mode of action, regulation of biosynthesis, and how these findings advance our understanding of reproductive neuroendocrinology.

## History of Neuroendocrinology as a Field

The discovery of “neurosecretion” in the first half of the twentieth century led to the creation of neuroendocrinology, a new research field of endocrinology. In the 1920s, Ernst and Berta Scharrer first proposed the new concept of neurosecretion, suggesting that hypothalamic neurons terminating in the neurohypophysis secrete neurohormones analogous to those of endocrine glands. This seminal idea was not accepted easily by the scientific community, leading to marked criticism, including the comments like: “We have just heard some very interesting things, … and also a great deal of nonsense.” In 1949, however, Bargmann established the concept of neurosecretion proposed by Ernst and Berta Scharrer. Subsequently, hypothalamic neuropeptides, oxytocin (1) and vasopressin (2), were identified to be neurohormones that are secreted from the neurohypophysis.

The morphology of hypothalamic neurons that terminate at the median eminence (ME) made Harris (3) to hypothesize that these hypothalamic neurons may secrete neurohormones from the ME into the hypophysial portal system to regulate the secretion of anterior pituitary hormones. Harris further summarized the first map showing different areas in the hypothalamus responsible for various pituitary hormone release, identified by contemporary lesions and electrical stimulation studies [(4), reviewed in Ref. (5)]. Halasz et al. (6) also contributed to the identification of the hypophysiotrophic area in the hypothalamus by pituitary transplantation method (5). McCann and Ramirez were the first to demonstrate the biological existence of luteinizing hormone (LH)-releasing factor (LHRF) in the basal middle hypothalamus [see Ref. (7) for a review]. Subsequently, the groups of Schally and Guillemin identified the structure of several neurohormones that regulate anterior pituitary hormone release, including thyrotropin-releasing hormone (TRH) (8, 9), gonadotropin-releasing hormone (GnRH) (10, 11), and growth hormone-inhibiting hormone (somatostatin) (12), in the brain of mammals. Schally and Guillemin were awarded a Nobel Prize in 1977 for the discoveries of these neurohormones. At the same time, Yalow also received this prize for her role in the development of radioimmunoassay that permitted the measurement of these neurohormones.

As described above, Schally’s (10) and Guillemin’s (11) groups discovered GnRH, a hypothalamic neuropeptide that stimulates the release of both LH and follicle-stimulating hormone (FSH) from gonadotropes in the anterior pituitary, in mammals. Subsequently, several GnRHs have been identified in other vertebrates (13–16). Although McCann et al. (17) suggested differential hypothalamic control mechanism of FSH secretion, it had been generally accepted that GnRH is the only hypothalamic neuropeptide regulating gonadotropin release in mammals and other vertebrates.

In 2000, however, Tsutsui and colleagues challenged this notion with the discovery of gonadotropin-inhibitory hormone (GnIH), a hypothalamic neuropeptide that actively inhibits gonadotropin release, in quail (18). Subsequent studies conducted by Tsutsui and colleagues over the past decade and a half demonstrated that GnIH is highly conserved among vertebrates, from agnathans to humans, acting as a key player regulating reproduction [for reviews, see Ref. (19–30)]. Recent studies by Tsutsui’s group have demonstrated that GnIH has important functions beyond the control of reproduction (31, 32). Based on these findings, it now appears that GnIH not only acts on the pituitary but in the brain to affect a number of behaviors, including reproductive behavior through changes in neurosteroid, such as neuroestrogen, biosynthesis in the brain [(32), for a review, see Ref. (33)]. Thus, the following 15 years of GnIH research in collaboration with world’s leading laboratories have permitted a more complete understanding of the neuroendocrine control of reproductive behavior and physiology [for reviews, see Ref. (19–22, 24–29, 34)].

Herein, this review summarizes the discovery of GnIH and the contribution of GnIH research over the past decade and a half, focusing on its mode of action, regulation of biosynthesis, and the ways that such contributions have contributed to the field of reproductive neuroendocrinology. This review also highlights the commonalities and diversity of GnIH structures and actions as well as the evolutionary origin of GnIH in chordates.

## Discovery of GnIH and Its Role in Reproduction

Gonadotropin-inhibitory hormone was discovered in the brain of the Japanese quail while searching for a novel peptide possessing a C-terminal sequence Arg-Phe-NH_2_ (RFamide peptide) in vertebrates (18). The first identification of an RFamide peptide dates back to the late 1970s, when Price and Greenberg purified a peptide Phe–Met–Arg–Phe–NH_2_ (FMRFamide) from the ganglia of the venus clam (35). Since this initial discovery, various RFamide peptides that act as neurotransmitters, neuromodulators, and peripheral hormones had been identified in invertebrates. However, immunohistochemical studies in vertebrates suggested the presence of an unknown hypothalamic RFamide peptide(s) that may regulate the secretion of anterior pituitary hormones (36, 37). Based on this information, Tsutsui’s laboratory searched for novel RFamide peptide(s) in the brain of the Japanese quail.

**Figure 1:**
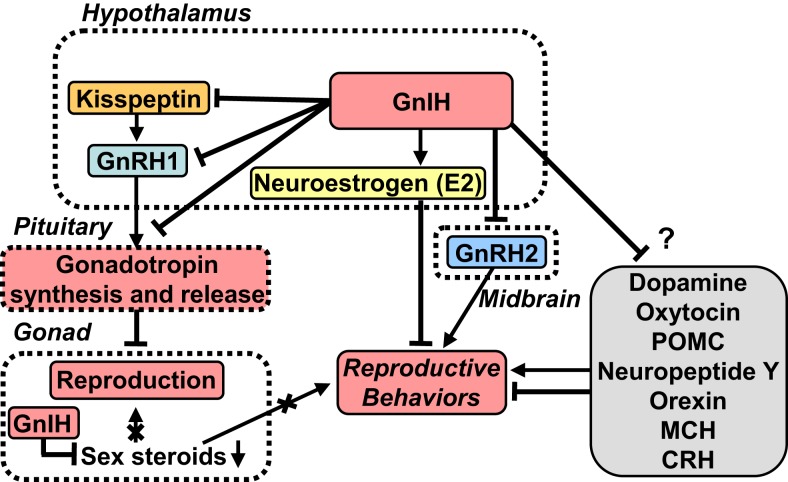
**A schematic model of GnIH actions on the regulation of reproduction and reproductive behaviors**. GnIH is a newly discovered hypothalamic neuropeptide that inhibits gonadotropin release in the quail brain (18). GnIH is highly conserved among vertebrates. GnIH acts as a key player in the regulation of reproduction and reproductive behaviors across vertebrates. Cell bodies for GnIH neurons are localized in the paraventricular nucleus (PVN) in birds and in the dorsomedial hypothalamic area (DMH) in mammals. Terminals from GnIH neurons are localized to the median eminence (ME) and GnRH1 neurons in the preoptic area (POA) in birds and mammals. GnIH receptor is expressed in gonadotropes in the pituitary and GnRH1 neurons in the POA in birds and mammals. Thus, GnIH inhibits gonadotropin synthesis and release by directly acting on gonadotropes in the pituitary and by inhibiting the activity of GnRH1 neurons in the POA via GnIH receptor in birds and mammals. GnIH neurons project not only to GnRH neurons but also to kisspeptin neurons in the hypothalamus in mammals. Kisspeptin neurons express GnIH receptor in mammals. GnIH and GnIH receptor are also expressed in steroidogenic cells and germ cells in gonads, and GnIH possibly acts in an autocrine/paracrine manner to suppress gonadal steroid production and germ cell differentiation and maturation in birds and mammals. GnIH participates not only in neuroendocrine functions but also in behavioral control in birds and mammals. GnIH inhibits reproductive behaviors, such as sexual and aggressive behaviors, and stimulates feeding behavior by acting within the brains of birds and mammals. Furthermore, GnIH inhibits reproductive behaviors by changing the biosynthesis of neuroestrogen (E2) in the POA. GnIH neurons further project to many other neurons in the brain suggesting multiple actions of GnIH. See the text for details.

A breakthrough occurred in 2000 when Tsutsui and colleagues discovered a novel RFamide peptide Ser–Ile–Lys–Pro–Ser–Ala–Tyr–Leu–Pro–Leu–Arg–Phe–NH_2_ (SIKPSAYLPLRFamide) that actively inhibited gonadotropin release in quail pituitary, providing the first demonstration of a hypothalamic neuropeptide inhibiting gonadotropin release in any vertebrate (18) (Table 1). Given its functional role, this neuropeptide was named GnIH (18) (Figure 1). In birds, cell bodies and terminals of GnIH neurons are located in the paraventricular nucleus (PVN) and ME, respectively (18). The C-terminal structure of GnIH is identical to chicken LPLRFamide which is the first reported RFamide peptide in vertebrates (38), but this peptide is likely to be a degraded fragment of chicken GnIH [(39), for reviews, see Ref. (20, 26, 27)] (Table 1). Subsequently, a cDNA encoding the precursor polypeptide for GnIH was identified in quail (40) and other avian species, such as chickens, sparrows, starlings, and zebra finches [for reviews, see Ref. (20, 26, 27)]. The GnIH precursor encompasses one GnIH and two GnIH-related peptides (GnIH-RP-1 and GnIH-RP-2) possessing an LPXRFamide (X = L or Q) motif at their C-terminus in all avian species studied. GnIH was further identified as a mature peptide in starlings (41) and zebra finches (42), and GnIH-RP-2 was identified in quail (40) (Table 1).

**Table 1:** **Molecular structure of mature GnIH peptides in chordates**.

Chordates	Peptide name	Molecular structure	Reference
Mammals	*human RFRP-1*	MPHSFANLPLRFa	Ubuka et al. (43)
	*human RFRP-3*	VPNLPQRFa	Ubuka et al. (43)
	*macaque RFRP-3*	SGRNMEVSLVRQVLNLPQRFa	Ubuka et al. (44)
	*bovine RFRP-1*	SLTFEEVKDWAPKIKMNKPVVNKMPPSAANLPLRFa	Fukusumi et al. (45)
	*bovine RFRP-3*	AMAHLPLRLGKNREDSLSRWVPNLPQRFa	Yoshida et al. (46)
	*rat RFRP-3*	ANMEAGTMSHFPSLPQRFa	Ukena et al. (47)
	*Siberian hamster RFRP-1*	SPAPANKVPHSAANLPLRFa	Ubuka et al. (48)
	*Siberian hamster RFRP-3*	TLSRVPSLPQRFa	Ubuka et al. (48)
Birds	*quail GnIH*	SIKPSAYLPLRFa	Tsutsui et al. (18)
	*quail GnIH-RP-2*	SSIQSLLNLPQRFa	Satake et al. (40)
	*chicken GnIH*	SIRPSAYLPLRFa	McConn et al. (39)
	*European starling GnIH*	SIKPFANLPLRFa	Ubuka et al. (41)
	*zebra finch GnIH*	SIKPFSNLPLRFa	Tobari et al. (42)
Reptiles	*red-eared slider GnIH*	SIKPVANLPLRFa	Ukena et al. (49)
	*red-eared slider GnIH-RP-1*	STPTVNKMPNSLANLPLRFa	Ukena et al. (49)
	*red-eared slider GnIH-RP-2*	SSIQSLANLPQRFa	Ukena et al. (49)
Amphibians	*bullfrog GRP/R-RFa*	SLKPAANLPLRFa	Koda et al. (50) and Chartrel et al. (51)
	*bullfrog GRP-RP-1*	SIPNLPQRFa	Ukena et al. (52)
	*bullfrog GRP-RP-2*	YLSGKTKVQSMANLPQRFa	Ukena et al. (52)
	*bullfrog GRP-RP-3*	AQYTNHFVHSLDTLPLRFa	Ukena et al. (52)
	*red-bellied newt LPXRFa-1*	SVPNLPQRFa	Chowdhury et al. (53)
	*red-bellied newt LPXRFa-2*	MPHASANLPLRFa	Chowdhury et al. (53)
	*red-bellied newt LPXRFa-3*	SIQPLANLPQRFa	Chowdhury et al. (53)
	*red-bellied newt LPXRFa-4*	APSAGQFIQTLANLPQRFa	Chowdhury et al. (53)
Teleost fishes	*goldfish LPXRFa-3*	SGTGLSATLPQRFa	Sawada et al. (54)
Agnathans	*sea lamprey LPXRFa-1a*	SGVGQGRSSKTLFQPQRFa	Osugi et al. (55)
	*sea lamprey LPXRFa-1b*	AALRSGVGQGRSSKTLFQPQRFa	Osugi et al. (55)
	*sea lamprey LPXRFa-2*	SEPFWHRTRPQRFa	Osugi et al. (55)
Protochordates	*amphioxus PQRFa-1*	WDEAWRPQRFa	Osugi et al. (56)
	*amphioxus PQRFa-2*	GDHTKDGWRPQRFa	Osugi et al. (56)
	*amphioxus PQRFa-3*	GRDQGWRPQRFa	Osugi et al. (56)

*Only structurally determined mature endogenous peptides are shown*.

Gonadotropin-inhibitory hormone is considered to be a key neuropeptide inhibiting avian reproduction because GnIH inhibits gonadotropin release in most avian species studied [for reviews, see Ref. (20, 26, 27)] (Figure 1). To demonstrate the biological mode of action of GnIH *in vivo*, Ubuka et al. (57) treated mature male quail with chronic GnIH. Chronic GnIH treatment decreases plasma LH concentration and the expressions of the common α, LHβ, and FSHβ subunit mRNAs. Additionally, Ubuka et al. (57) established that GnIH treatment induces testicular apoptosis and decreases the diameter of seminiferous tubules and testosterone concentration in mature birds. In immature birds, GnIH treatment suppresses normal testicular growth and testosterone concentration (57). Thus, GnIH appears to suppress gonadal development and maintenance by decreasing gonadotropin synthesis and release in birds (Figure 1).

To determine if these findings extend to mammals, including humans, Tsutsui and colleagues sought to identify GnIH in the hypothalamus of mammals and primates (43, 44, 47, 48, 58). All the identified and putative mammalian and primate GnIH peptides possess a common C-terminal LPXRFamide (X = L or Q) motif, like avian GnIH and GnIH-RPs [for reviews, see Ref. (20, 21, 26–29)] (Table 1). Therefore, these GnIH peptides were designated as LPXRFamide peptides on the basis of their structures. Mammalian and primate GnIH peptides are also called RFamide-related peptide 1 and 3 (RFRP-1 and -3) (Table 1). Kriegsfeld et al. (58) found that *in vivo* administration of avian GnIH centrally or peripherally to female Syrian hamsters inhibits LH release (58). Central administration of hamster GnIHs (RFRP-1 and -3) also inhibits LH release in Siberian hamsters (48). Central administration of rat GnIH (RFRP-3) to male rats also inhibits LH release (59) and GnRH-elicited gonadotropin release (60, 61). Reduction in LH pulse amplitude and inhibition of GnRH-elicited gonadotropin release and synthesis by mammalian GnIH (RFRP-3) have also been reported in ovine (62, 63) and bovine (64). Because the structure of human GnIH (RFRP-3) is the same as ovine GnIH (RFRP-3) (43), the hypohysiotropic action of human/ovine GnIH (RFRP-3) was assessed in ovine pituitary in collaboration with the Clarke laboratory. Human/ovine GnIH (RFRP-3) inhibits GnRH-stimulated secretion of both LH and FSH (62) demonstrating that, as with avian GnIH, mammalian and primate GnIH inhibit gonadotropin release and synthesis and GnRH-elicited gonadotropin secretion [for reviews, see Ref. (19–21, 26–29)] (Figure 1).

## Commonalities and Diversity of GnIH Structures and Actions

To place these findings into a broader perspective, Tsutsui and colleagues further identified GnIH peptides in the brain of reptiles, amphibians, and fish. All of the identified and putative GnIHs in these vertebrates also possess a common C-terminal LPXRFamide (X = L or Q) motif, like avian, mammalian, and primate GnIHs (49–54, 65, 66) (Table 1). Thus, GnIH peptides exist in the brain of vertebrates from fish to humans [for reviews, see Ref. (20–30)]. Goldfish GnIH precursor cDNA encodes three GnIHs, gfLPXRFa-1, -2, and -3 (54). These goldfish GnIH peptides (gfLPXRFa-1, -2, and -3) have both inhibitory and stimulatory effects on gonadotropin synthesis and release, possibly depending on reproductive conditions (67–70). Zebrafish GnIH peptide, zfLPXRF-3, also has an inhibitory effect on gonadotropin release (71).

As mentioned above, GnIH peptides were identified in representative species of gnathostomes. However, GnIH peptide had not been identified in agnathans, the most ancient lineage of vertebrates (72). Therefore, Tsutsui and colleagues searched for agnathan GnIH in collaboration with the Sower and Nozaki laboratories (55). Synteny analysis showed the existence of the gene for GnIH in sea lamprey, and Osugi et al. (55) cloned lamprey GnIH precursor cDNA that encodes GnIH peptides. Subsequently, three mature GnIH peptides were identified from the brain of sea lamprey by immunoaffinity purification and mass spectrometry (55) (Table 1). The identified lamprey GnIHs share a common C-terminal PQRFamide motif (55), unlike GnIHs identified of gnathostomes. However, phylogenetic analysis showed that the identified lamprey GnIH precursor groups with the LPXRFa peptide precursors of vertebrates, whereas the previously identified lamprey PQRFa peptide precursor (73) groups with the PQRFa peptide precursors of vertebrates. Accordingly, we concluded that the lamprey GnIH precursor gene is the ortholog of LPXRFa peptide gene (55).

Lamprey GnIH neurons are located in the hypothalamus (55) with immunoreactive fibers projecting to GnRH3 neurons (55). Few lamprey GnIH immunoreactive fibers were observed in the neurohypophysis compared to abundant lamprey GnRH3 immunoreactive fibers (55). Osugi et al. (55) then analyzed the effects of lamprey GnIH peptides on the expressions of lamprey GnRHs and the gonadotropin β subunit. It was found that one of the lamprey GnIH peptides increases the expressions of lamprey GnRH3 and gonadotropin β RNA (55). Thus, GnIH is present in the brain of lamprey, the oldest lineage of vertebrates, and may act on GnRH3 neurons to stimulate the expression of gonadotropin β in the pituitary (55). Based on these findings, it is speculated that GnIH emerged in agnathans as a stimulatory neuropeptide and changed into an inhibitory neuropeptide during vertebrate evolution.

## Evolutionary Origin of GnIH

The C-terminal structure of most GnIH peptides is LPXRFamide (X = L or Q), making them a member of the RFamide peptide family [for reviews, see Ref. (20–30)]. Four more groups, i.e., the neuropeptide FF (NPFF; PQRFamide peptide) group, the kisspeptin group, the pyroglutamylated RFamide peptide (QRFP)/26RFamide group, and the prolactin-releasing peptide (PrRP) group, have been documented in vertebrates [for reviews, see Ref. (20, 21, 23, 30)]. Because the C-terminal structure of GnIH peptides is similar to NPFF peptides that have a C-terminal PQRFamide motif, further clarification of the NPFF peptide gene in agnathans was warranted. NPFF is a neuropeptide involved in pain modulation [for a review, see Ref. (74)]. Accordingly, Tsutsui and colleagues sought to identify the cDNAs of NPFF peptides in the brain of lamprey and hagfish (73, 75). Phylogenetic analysis established that agnathans possess both GnIH and NPFF precursor genes. Agnathan NPFF peptides were further identified in sea lamprey and hagfish. The identified agnathan NPFF peptides had the same C-ternimal PQRFamide motif as agnathan GnIH peptides (73, 75).

The findings that agnathans have both GnIH and NPFF genes and their mature peptides have the same C-terminal PQRFamide motif (55, 73, 75) suggest that the GnIH and NPFF genes were derived from a common ancestral gene in protochordates. To test this hypothesis, Tsutsui and colleagues identified an amphioxus PQRFamide peptide precursor cDNA that encodes three putative PQRFamide peptides (56). Subsequently, three endogenous amphioxus PQRFamide peptides were identified by immunoaffinity purification and mass spectrometry (56) (Table 1). Phylogenetic analysis showed that the amphioxus PQRFamide peptide precursor occurs before the divergence between the GnIH and NPFF groups in vertebrates (56). Synteny analysis showed that the conserved synteny region exists around the loci of the amphioxus PQRFamide peptide gene, GnIH gene, and NPFF gene in vertebrates (56). The amphioxus PQRFamide peptide gene is located near the HOX cluster, whereas the GnIH and NPFF genes in vertebrates are located near the HOXA and HOXC clusters, respectively, suggesting that the GnIH and NPFF genes may have duplicated through whole-genome duplications (56). Accordingly, the amphioxus PQRFamide peptide gene is considered to be close to the ancestor of the GnIH and NPFF genes (56, 76). Thus, the GnIH and NPFF genes may have diverged from a common ancestral gene in the protochordate through whole-genome duplication event during vertebrate evolution.

## Mode of GnIH Action on Gonadotropin Secretion

### Identification of GnIH Receptor

To investigate the mode of action of GnIH on gonadotropin secretion in birds, Tsutsui and colleagues identified the receptor for GnIH in quail. They identified GnIH receptor as GPR147, a member of the G-protein-coupled receptor (GPCR) superfamily (77), which is also called NPFF receptor 1 (NPFF1). Membrane fraction of COS-7 cells that are transfected with GnIH receptor cDNA binds with high affinity to GnIH and GnIH-RPs (77). Since GnIH receptor is expressed in gonadotropes in the anterior pituitary, GnIH can act directly on gonadotropes to reduce gonadotropin release in birds [for reviews, see Ref. (19–21, 26–29, 34)] (Figure 1). In addition to acting on the anterior pituitary, GnIH neurons project to GnRH1 neurons (41, 78) that express GnIH receptor (41) (Figure 1). Thus, GnIH not only acts on gonadotropes but also acts on GnRH1 neurons to inhibit gonadotropin synthesis and release in birds [for reviews, see Ref. (19–21, 26–29, 34)] (Figure 1).

In mammals, Hinuma et al. (79) identified a specific receptor for mammalian GnIH, RFRP, which is identical to GPR147 and named it OT7T022 by the reverse pharmacological approach. In the human genome, there are approximately 700 GPCR genes, and the receptors whose ligands are still unknown are called orphan receptors. Hinuma et al. (79) searched for specific receptors for ligands by testing whether (1) increases in calcium ions, (2) increases in cAMP, or (3) decreases in cAMP happens in the cells transfected with the receptor by ligand stimulation [for a review, see Ref. (80)]. In the same year, Bonini et al. (81) reported two GPCRs for NPFF and designated them as NPFF1 (identical to GPR147) and NPFF2 (identical to GPR74). Bonini et al. (81) cloned these receptors by GPCR-targeted degenerate PCR using rat genomic DNA. As mentioned previously, it is thought that the GnIH (LPXRFamide peptide) and NPFF (PQRFamide peptide) genes have diverged from a common ancestral gene through gene duplication (55, 56, 76). It is also thought that GPR147 and GPR74 are paralogous from synteny analysis (82) and phylogenetic analysis (83). Analyses of binding affinities of GnIH and NPFF for GPR147 and GPR74 and their signal transduction pathways reveal that GnIH has a higher affinity for GPR147, whereas NPFF has potent agonistic activity for GPR74 (46, 81, 84), suggesting that GPR147 (NPFF1, OT7T022) is the primary receptor for GnIH.

### GnIH Cell Signaling

To further understand the cellular cascade by which GnIH impacts gonadotropes in the anterior pituitary, Tsutsui and colleagues investigated GnIH signaling pathways in the mouse gonadotrope cell line, LβT2. First, the expression of GnIH receptor mRNA in LβT2 cells was shown by RT-PCR (85). Subsequently, the inhibitory effects of GnIH on GnRH-induced signaling pathways were demonstrated; mouse GnIHs effectively reduce GnRH-induced cAMP production and extracellular signal-regulated kinase (ERK) phosphorylation (85). Furthermore, mouse GnIHs reduce GnRH-induced LHβ expression and LH release (85). The stimulatory effect of GnRH on gonadotropin expression is suppressed by adenylate cyclase (AC) and protein kinase A (PKA) inhibitors but not by protein kinase C (PKC) inhibitor (85). Accordingly, mouse GnIH reduces GnRH-stimulated gonadotropin secretion by specifically interfering with GnRH actions via a AC/cAMP/PKA-dependent ERK pathway (85).

Following the discovery of GnIH, kisspeptin was discovered in mammals. Opposite to GnIH, kisspeptin has a stimulatory effect on GnRH neurons and the hypothalamic–pituitary–gonadal axis (HPG axis) in mammals (86–89). GnIH neurons project not only to GnRH1 neurons in the preoptic area (POA) but also to kisspeptin neurons in the hypothalamus and may regulate their activities [for reviews, see Ref. (19–21, 26–29, 90)] (Figure 1). GnIH neurons project to GnRH2 neurons and many other neurons suggesting multiple actions of GnIH [for reviews, see Ref. (19–21, 26–29)] (Figure 1).

## Regulation of GnIH Biosynthesis in the Brain by Environmental and Internal Factors

### Influence of Photoperiod Mediated by Melatonin

Investigating the regulatory mechanisms of GnIH expression in the brain has important implications for understanding the physiological role of GnIH. Photoperiodic mammals regulate reproductive activities according to the annual cycle of changes in the nocturnal secretion of melatonin (91). There is also evidence in birds that melatonin is involved in the regulation of several seasonal processes including gonadotropin secretion and gonadal activity (92–95), despite the accepted dogma that birds do not use seasonal changes in melatonin secretion to time their reproductive effort (96, 97).

**Figure 2:**
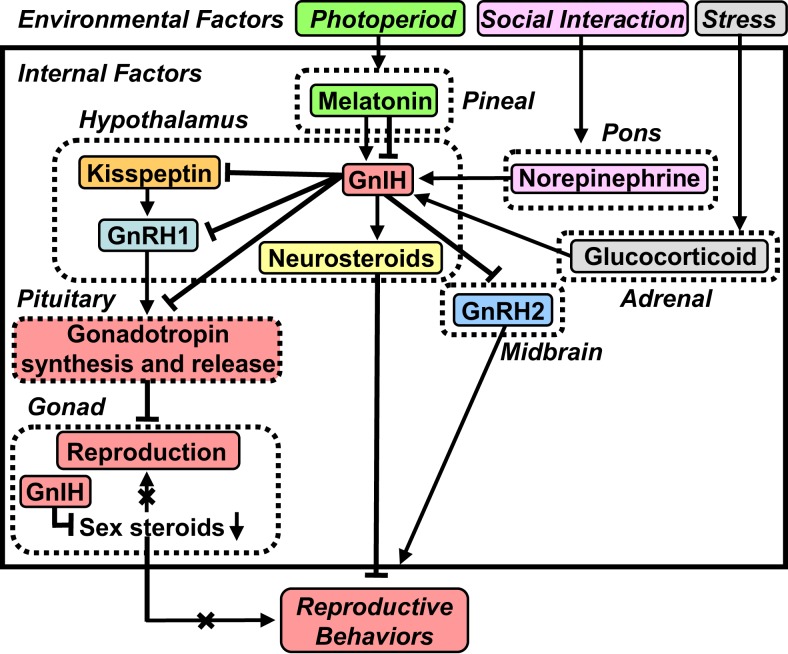
**A schematic model of the neuroendocrine integration of environmental factors and internal factors to control GnIH expression and release**. The neuroendocrine integration of environmental factors, such as photoperiod, stress, and social interaction, and internal factors, such as GnIH, melatonin, glucocorticoid, and norepinephrine (NE), is important for the control of reproduction and reproductive behaviors. GnIH inhibits the expression and release of gonadotropins and the expression of reproductive behaviors in birds and mammals. GnIH expression and release are photoperiodically modulated via a melatonin-dependent process in birds and mammals. Melatonin increases GnIH expression in quail and rats, whereas melatonin decreases GnIH expression in hamsters and sheep. Stress increases GnIH expression mediated by the actions of glucocorticoids in birds and mammals. GnIH may be a mediator of stress-induced reproductive disruption. The social environment also changes GnIH expression and release mediated by the action of NE. See the text for details.

To explore whether or not GnIH is part of the mechanism driving melatonin-induced seasonal changes in reproduction, Tsutsui and colleagues investigated the action of melatonin on the expression of GnIH in quail, a highly photoperiodic bird species. Initial findings demonstrated that melatonin induces GnIH expression in birds (Figure 2). More specifically, Ubuka et al. (98) first found that melatonin removal by pinealectomy, combined with orbital enucleation (Px plus Ex), decreases the expressions of GnIH mRNA and GnIH peptide labeling in the brain of quail (98). Ubuka et al. (98) further found that melatonin administration increases the expressions of GnIH mRNA and GnIH peptide in the brain of quail (98). Importantly, they found that Mel_1c_, a melatonin receptor subtype, is expressed in GnIH neurons in quail (98). These findings established that melatonin acts directly on GnIH neurons to induce GnIH expression in this species (Figure 2). Chowdhury et al. (99) further demonstrated that melatonin not only increases GnIH expression but also increases GnIH release in quail (Figure 2). Interestingly, GnIH release is photoperiodically controlled in quail with diurnal changes that are negatively correlated with plasma LH concentration (99). As one would expect, GnIH release increases under short day (SD) photoperiods, when the duration of nocturnal secretion of melatonin increases (99). Together, these findings indicate that melatonin derived from the pineal gland and eyes acts directly on GnIH neurons via Mel_1c_ to induce GnIH expression and release in birds (29, 98–101) (Figure 2).

In contrast to the results seen in quail, melatonin reduces GnIH expression in Syrian and Siberian hamsters, both photoperiodic mammals (48, 102, 103) (Figure 2). Specifically, GnIH mRNA levels and the number of GnIH immunoreactive cell bodies are reduced in sexually quiescent Syrian and Siberian hamsters exposed to SD photoperiods, compared to sexually active animals maintained under long day (LD) photoperiods. These photoperiodic changes in GnIH expression are abolished in Px hamsters, and injections of LD hamsters with melatonin reduce the expression of GnIH to SD levels (48, 102). Analogous seasonal patterns of GnIH expression have been observed in European (104) and Turkish (105) hamsters as well as the semidesert rodent, Jerboa (106). Although these results are suggestive of a role for GnIH in seasonal breeding, they are inconsistent with a straightforward model of seasonal reproductive control by this peptide. One possibility is that hamsters require enhanced GnIH expression to suppress GnRH during the initial period of regression, whereas this level of inhibition is not necessary in hamsters with a fully regressed reproductive axis and low testosterone (T) concentrations. Another possibility emerged from our findings in male Siberian hamsters. In this species, GnIH administration suppresses HPG axis function in LD, reproductive-competent animals but stimulates gonadotropin secretion in SD, reproductively quiescent animals (48), indicating that the impact of GnIH may depend on reproductive status or season. There are also reports showing that the expression of GnIH is regulated by melatonin and season in sheep (107, 108) and rats (109). Thus, as in quail, the expression of GnIH is photoperiodically modulated via a melatonin-dependent process in mammals (Figure 2).

We still do not have data as to why melatonin stimulates GnIH expression in quail (98) and inhibits GnIH expression in hamsters (48, 102) and other mammals. Although expression of melatonin receptor in GnIH neurons is still not demonstrated in mammals, one possibility is that melatonin triggers different intracellular signals in GnIH neurons in quail because mammals do not have Mel_1c_ melatonin receptor subtype (110). Another possibility is that melatonin indirectly regulates GnIH expression in mammals unlike quail. These possibilities should be tested in future studies.

### Influence of Stress Mediated by Glucocorticoids

It is well known that stress can reduce reproduction across vertebrates (111). To explore whether stress changes GnIH expression in birds, Calisi et al. (112) investigated the effect of capture-handling stress on GnIH expression in male and female adult house sparrows. Calisi et al. (112) found that more GnIH-positive neurons are observed in fall birds versus those sampled in the spring, and GnIH-positive neurons are increased by capture-handling stress in spring birds. These observations indicate that stress influences GnIH during the breeding season (112). These findings suggested that stress may act through GnIH neurons to inhibit reproductive function in birds.

In mammals, Kirby et al. (113) showed that both acute and chronic immobilization stress lead to an upregulation of the expression of GnIH in the dorsomedial hypothalamic area (DMH) of male rats associated with inhibition of downstream hypothalamic–pituitary–testicular activity (Figure 2). Adrenalectomy blocks this stress-induced increase in GnIH expression. Immunohistochemistry revealed that GnIH neurons express glucocorticoid receptor (GR), suggesting that adrenal glucocorticoids act directly on GnIH neurons to increase GnIH expression (Figure 2). Together, these observations indicate that GnIH is an important integrator of stress-induced suppression of reproductive function in mammals (113).

Recently, Son et al. (114) demonstrated that GR mRNA is expressed in GnIH neurons in the PVN of quail, suggesting that glucocorticoids can directly regulate GnIH transcription (Figure 2). It was also found that treatment with corticosterone (CORT) increases GnIH mRNA expression in the quail diencephalon (114) (Figure 2). Subsequently, Son et al. (114) investigated the mechanism of activation of GnIH transcription by CORT using a GnIH-expressing neuronal cell line, rHypoE-23, derived from rat hypothalamus. Importantly, GR mRNA is expressed in rHypoE-23 cells, and CORT treatment increases GnIH mRNA expression (114). Son et al. (114) further found that CORT stimulates GR recruitment to the GC-response element (GRE) present in the rat GnIH promoter region, providing further support that CORT induces GnIH expression via GR in GnIH neurons (Figure 2). Taken together, it appears that stress reduces gonadotropin release, at least in part, through an increase in GnIH expression. More recent evidence also indicates that GnIH might itself regulate the stress response in mice (115).

### Influence of Social Interactions Mediated by Norepinephrine

In addition to the regulation of GnIH expression by environmental factors, photoperiod, and stress, the social environment may influence the GnIH system (Figure 2). To examine this possibility, Calisi et al. (116) investigated the impact of mating competition on GnIH. Nesting opportunities for pairs of European starlings were manipulated, and GnIH mRNA and GnIH content as well as GnRH content in the brain were examined. Birds with nest boxes have fewer numbers of GnIH-producing cells than those without nest boxes. However, GnRH content does not vary with nest box ownership. These observations suggest that GnIH may serve as a modulator of reproductive function in response to social environment (116).

Reproductive physiology and behavior are variable, both within and between individuals. It is known that the presence of a female bird as well as copulation rapidly decrease plasma T concentrations in male quail (117, 118). Based on these earlier observations, Tsutsui and colleagues investigated the neurochemical mechanism by which social stimuli alter reproductive physiology and behavior (Figure 2). Tobari et al. (31) first found that norepinephrine (NE) release increases rapidly in the PVN of quail when viewing a female conspecific (Figure 2). Likewise, GnIH mRNA expression increases in the PVN, with associated decreases in LH concentrations in plasma, when males view a female (Figure 2). Tobari et al. (31) then established a link between these two events by showing that NE application stimulates GnIH release from diencephalic tissue blocks *in vitro*. Double-label immunohistochemistry revealed that GnIH neurons are innervated by noradrenergic fibers and immunohistochemistry combined with *in situ* hybridization demonstrated that GnIH neurons expressed α2A-adrenergic receptor mRNA. Together, these observations indicate that female presence increases NE release in the PVN and stimulates GnIH release, resulting in the suppression of LH release in quail (31) (Figure 2).

## Multiple Actions of GnIH

### Direct Regulation of Gonadal Activity

The aforementioned findings indicate that GnIH is a key player in the regulation of reproduction across vertebrates, reducing gonadotropin synthesis and release by decreasing the activity of GnRH1 neurons and decreasing the activity of pituitary gonadotropes, inevitably suppressing gonadal steroid secretion and spermatogenesis (Figure 1). In addition to these central actions of GnIH, several lines of evidence point to direct, local regulation of gonadal activity [for reviews, see Ref. (19, 20, 26–29, 32, 34)] (Figure 1). GnIH and GnIH receptor are expressed in steroidogenic cells and germ cells in the gonads of birds and mammals (119–125), with GnIH possibly acting in an autocrine/paracrine manner to suppress gonadal steroid production and germ cell differentiation and maturation (119–125) (Figure 1). There is also evidence in songbirds that gonadal GnIH responds directly to melatonin, metabolic challenge, and cues of stress in a seasonal manner (126–128)

### Regulation of Feeding Behavior

It is becoming clear that GnIH participates not only in neuroendocrine functions but also in behavioral control. In environments where energy availability fluctuates, animals use photoperiod to phase breeding with anticipated times of maximal food availability (129). Should food become scarce during the breeding season, reproduction is temporarily inhibited (130, 131). Food deprivation and other metabolic challenges inhibit reproductive axis functioning and sexual motivation (132–136). GnIH may relay metabolic information to the HPG axis and regulate neural feeding circuits [for a review, see Ref. (19)].

Tachibana et al. (137) showed that intracerebroventricular (ICV) injections of GnIH, GnIH-RP-1, and GnIH-RP-2 stimulate food intake in chicks (137). In further support of a stimulatory role for GnIH in feeding, anti-GnIH antiserum suppresses appetite induced by fasting but does not modify feeding under *ad libitum* conditions (137). Similarly, Fraley et al. (138) reported that ICV injection of GnIH, but not of GnIH-RP1, suppresses plasma LH and stimulates feeding in adult Pekin ducks. To establish the neurochemical cascade underlying the actions of GnIH on feeding, Tachibana et al. (139) explored the possibility that the orexigenic effect of GnIH occurs via actions on the opioid and nitric oxide (NO) systems. It was found that the orexigenic effect of ICV injected GnIH is attenuated by coinjection of β-funaltrexamine (an opioid μ-receptor antagonist) but not ICI-174,864 (an opioid δ-receptor antagonist) and nor-binaltorphimine (an opioid κ-receptor antagonist) in chicks. It was also found that coinjection of a non-selective NO synthase inhibitor does not affect GnIH-induced feeding behavior (139). More recently, McConn et al. (39) investigated the central mechanism of the GnIH orexigenic response in chicks. It was found that neuropeptide Y (NPY) mRNA is increased, while pro-opiomelanocortin (POMC) mRNA is decreased in the hypothalamus following ICV injection of chicken GnIH. Additionally, ICV GnIH injections increase c-fos immunoreactive cells in the lateral hypothalamic area (LHA). McConn et al. (39) further found that in isolated LHA, melanin-concentrating hormone (MCH) mRNA is increased by ICV administration of GnIH. Together, these observations suggest that opioid μ-receptor-positive, NPY, POMC, and MCH neurons are likely involved in the GnIH orexigenic response.

In mammals, there are several reports indicating that ICV administration of GnIH increases food intake in rats (59) and sheep (140). Fu and van den Pol (141) showed that chicken GnIH and human GnIH inhibit POMC neurons and attenuate kisspeptin cell excitation by a mechanism based on opening potassium channels in mouse brain slices. Jacobi et al. (142) reported that GnIH inhibits the firing rate in POMC neurons and has a predominantly inhibitory effect on action potential activity in NPY neurons in mice. Jacobi et al. (142) also reported that NPY neurons have close contacts from GnIH fibers. Together, these observations indicate that GnIH participates not only in reproduction but also in feeding behavior in birds and mammals.

### Regulation of Reproductive Behaviors

Gonadotropin-inhibitory hormone also acts on the brain to regulate reproductive behaviors, such as sexual and aggressive behaviors (32, 143, 144) (Figure 1). For example, Bentley et al. (143) showed that a centrally administered physiological dose of GnIH inhibits copulation solicitation in estrogen-primed female white-crowned sparrows exposed to the song of males. It is known that GnRH2 enhances copulation solicitation in estrogen-primed female white-crowned sparrows exposed to the song of males (145). Because GnIH neurons terminate in close proximity of GnRH2 neurons and GnRH2 neurons express GnIH receptor in songbirds (41), GnIH may inhibit copulation solicitation by inhibiting GnRH2 neurons in female songbirds (143) (Figure 1). Ubuka et al. (144) directly investigated this possibility by applying RNA interference (RNAi) to the GnIH gene and examining the behavior of male and female white-crowned sparrows in collaboration with the Wingfield laboratory. GnIH RNAi reduces resting time, spontaneous production of complex vocalizations, and stimulates agonistic vocalizations. Additionally, GnIH RNAi enhances song production of short duration in male birds when they are challenged by playbacks of novel male songs. These observations indicate that GnIH gene silencing induces arousal. Ubuka et al. (144) further showed that the activities of male and female birds are negatively correlated with GnIH mRNA expression in the PVN. The density of GnIH neuronal fibers in the ventral tegmental area is decreased by GnIH RNAi in female birds, and the number of GnRH1 and GnRH2 neurons that receive close appositions of GnIH neuronal fiber terminals is negatively correlated with the activity of male birds (144) (Figure 1). Recently, Ubuka et al. (32) have demonstrated that GnIH also inhibits aggressive behavior in male quail. Thus, it is considered that GnIH decreases sexual and aggressive behaviors in birds [for reviews, see Ref. (19, 29, 34)] (Figure 1).

Similar results have been observed in mammals. For example, Johnson et al. (59) reported that ICV administration of GnIH suppresses male sex behavior in rats. Piekarski et al. (146) found that ICV administration of GnIH reduces sexual motivation and vaginal scent marking but not lordosis behavior in female hamsters. According to Piekarski et al. (146), GnIH administration alters fos expression in key neural loci implicated in female sexual behavior, including the medial POA, medial amygdala, and bed nucleus of the stria terminalis. These observations suggest that GnIH is an important modulator of female proceptive sexual behavior and motivation (Figure 1). Thus, as in birds, GnIH is not only in a position to regulate the HPG axis but may also act as a neuromodulator to drive the neural circuitry underlying socially motivated behavior.

### Regulation of Neurosteroidogenesis

**Figure 3:**
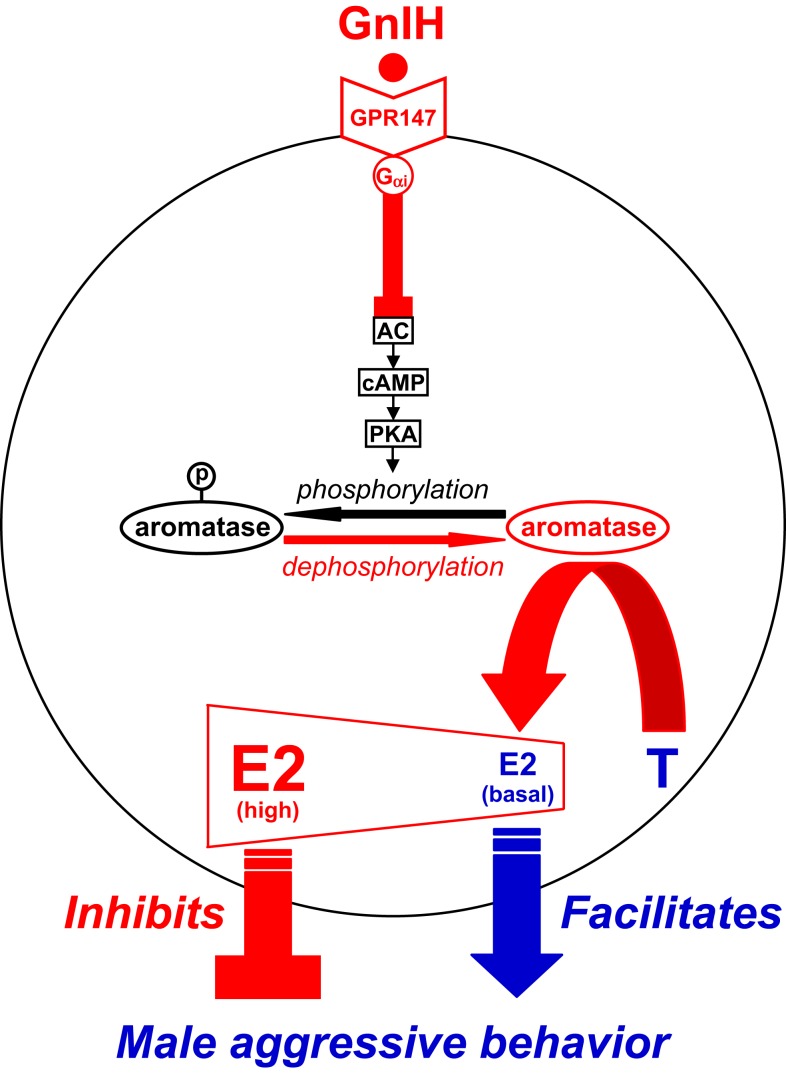
**Model of the intracellular mechanism of GnIH and its receptor (GPR147) that control male aggressive behavior by regulating the activity of aromatase and neuroestrogen synthesis in the brain**. GPR147 is expressed in aromatase immunoreactive cells in the brain. GPR147 is coupled to G_αi_ protein that inhibits the activity of adenylate cyclase (AC) and decreases cAMP production and the activity of protein kinase A (PKA). Inhibition of AC/cAMP/PKA pathway may decrease phosphorylated aromatase and increase dephosphorylated aromatase. 17β-Estradiol (E2) synthesized from testosterone (T) by aromatase in the brain especially in the preoptic area (POA) regulates male aggression. The administration of GnIH activates aromatase by decreasing phosphorylated aromatase and stimulates neuroestrogen synthesis in the brain. GnIH may inhibit aggressive behavior by directly activating aromatase and increasing neuroestrogen synthesis in the brain beyond its optimum concentration for the expression of aggressive behavior. Partially adapted from Ubuka and Tsutsui (33).

It is becoming increasingly clear that the interaction of neuropeptides and neurosteroids plays a role in the regulations of some brain functions [for a review, see Ref. (147)]. Recently, Ubuka et al. (32) found that GnIH activates cytochrome P450 aromatase (P450arom) and increases neuroestrogen synthesis in the avian brain (32) (Figures 1 and 3). Importantly, the actions of GnIH on neuroestrogen synthesis change the expression of aggressive behavior in birds (32) (Figures 1 and 3), providing a new concept that GnIH modifies the neurosteroid milieu in the brain to modulate aggressive behavior.

Further exploring the ability of GnIH to alter neurosteroid production has led to important insight into the control of aggressive behavior in quail. Sexually mature male quail frequently fight with intense aggressiveness, by using stereotyped actions (148, 149). Aggressive behavior of male quail is thought to be androgen dependent (148–150), but there is generally no correlation between aggressiveness and peripheral T concentration (150). It is also known that aggression in males is activated by aromatizable androgens, such as T and androstenedione (AD), but not by non-aromatizable androgens, such as dihydrotestosterone (DHT), and that administration of P450arom inhibitors blocks T-induced aggression (150, 151). Thus, the action of gonadal androgen on aggressive behavior requires its aromatization into estrogen (neuroestrogen) in the brain (152–154).

GnIH neurons project to the ME and other brain areas, such as the POA (18, 155, 156) and the periaqueductal central gray [PAG (41)] in birds. GnIH receptor is also expressed in the POA (41, 77) and PAG (41). These brain areas are known to regulate aggressive behavior (157, 158). The POA is also known to be the most critical site of aromatization of gonadal androgen by P450arom and neuroestrogen action for the activation of aggressive behavior in male quail (159, 160). Because GnIH decreases aggressive behavior in male birds (32, 144), GnIH may decrease this behavior by regulating P450arom activity and neuroestrogen synthesis in the brain (Figures 1 and 3). Ubuka et al. (32) therefore investigated whether GnIH neuronal fibers innervate P450arom cells and whether P450arom cells express GnIH receptor in the POA of male quail. It was found that abundant GnIH immunoreactive neuronal fibers are distributed in the vicinity of P450arom immunoreactive cells in the POA (32). It was also found that GnIH receptor is expressed in P450arom immunoreactive cells in the POA (32). Furthermore, GnIH stimulates P450arom activity and increases neuroestrogen synthesis in the POA through GnIH receptor (32) (Figures 1 and 3). These studies provided the first evidence that a hypothalamic neuropeptide can regulate neuroestrogen synthesis in the brain.

Importantly, the increase in neuroestrogen concentrations in the POA was associated with a decrease in aggressive behavior (32). Therefore, the effect of central administration of various doses of estradiol-17β (E2) on aggressive behavior was tested in male quail. Ubuka et al. (32) found that central administration of E2 at higher doses decreases aggressive behavior unlike E2 at lower doses (32). This observation indicates that the action of neuroestrogen is essential for the expression of aggressive behavior, but higher concentrations of neuroestrogen in the brain decrease this behavior. Taken together, GnIH decreases aggressive behavior by activating P450arom and increasing neuroestrogen synthesis in the brain beyond its optimum concentration for the expression of aggressive behavior of male birds (32) (Figures 1 and 3).

Ubuka et al. (32) further investigated the mode of action of GnIH on the stimulation of P450arom activity. There is important evidence that P450arom activity is not only controlled in the long term by transcription of the P450arom gene *Cyp19* by steroids, but also in the short term by phosphorylation by neurotransmitters, such as glutamate (161). Balthazart’s group demonstrated that P450arom activity in the hypothalamus of male quail is rapidly downregulated by phosphorylation (161–165). Therefore, GnIH may activate P450arom by dephosphorylation of phosphorylated P450arom. Ubuka et al. (32) found that ICV administration of GnIH reduces phosphorylated P450arom in the POA in the short term compared with control birds (32). Ubuka et al. (32) also found that the action of GnIH on neuroestrogen synthesis in the POA is abolished by concomitant administration of RF9 (166, 167), a potent antagonist of GnIH receptor, or fadrozole (168, 169), an inhibitor of P450arom. Based on these findings, it is apparent that GnIH stimulates neuroestrogen synthesis in the POA by activating P450arom through dephosphorylation after binding to GnIH receptor in P450arom cells (Figure 3).

## Conclusion and Future Directions

The discovery of GnIH in 2000 and the contributions aimed at understanding its evolutionary history and functions have markedly advanced the progress of reproductive neuroendocrinology. Studies on GnIH over the past decade and a half have demonstrated that GnIH is a key player in the regulation of reproduction across vertebrates. It now appears that GnIH acts on the pituitary and the brain to modulate the reproductive axis and reproductive behaviors (Figure 1). In this review, the commonalities and diversity of GnIH structures and actions and the evolutionary origin of GnIH in chordates were also highlighted. The discovery of GnIH has changed our understanding of the regulation of reproductive physiology and behavior. As a result, more than 50 laboratories worldwide are now working on GnIH.

Following the discovery of GnIH, kisspeptin was also discovered in mammals. GnIH and kisspeptin are both comparatively new members of the RFamide peptide family and act on the HPG axis to downregulate and upregulate the reproductive system, respectively. Thus, we now know that GnRH is not the only hypothalamic neuropeptide regulating reproduction. Importantly, GnIH neurons project not only to GnRH neurons but also to kisspeptin neurons in the hypothalamus (Figure 1). GnRH neurons and kisspeptin neurons express GnIH receptor. We expect that future studies will reveal previously unknown interactions among GnIH, GnRH, and kisspeptin (Figure 1). The fact that GnIH neurons project to many other neurons in the brain suggests multiple actions of GnIH that have not yet been discovered (Figure 1).

GnIH activates P450arom activity in the brain (Figure 1) and may change the formation of other neurosteroids by activating or inactivating their steroidogenic enzymes. Furthermore, steroidogenic enzymes are expressed not only in the brain but also in the pineal gland (170–173). Future studies are needed to further develop the emerging concept that hypothalamic neuropeptides may modify the neurosteroid milieu in the brain and pineal gland to impact their function.

## Conflict of Interest Statement

The authors declare that the research was conducted in the absence of any commercial or financial relationships that could be construed as a potential conflict of interest.
